# A ranking method for the concurrent learning of compounds with various activity profiles

**DOI:** 10.1186/s13321-014-0050-6

**Published:** 2015-01-16

**Authors:** Alexander Dörr, Lars Rosenbaum, Andreas Zell

**Affiliations:** Center for Bioinformatics Tübingen (ZBIT), University of Tuebingen, Sand 1, Tübingen, 72076 Germany

**Keywords:** Machine learning, Support vector machine, Ranking, Virtual screening, Multi-target

## Abstract

**Background:**

In this study, we present a SVM-based ranking algorithm for the concurrent learning of compounds with different activity profiles and their varying prioritization. To this end, a specific labeling of each compound was elaborated in order to infer virtual screening models against multiple targets. We compared the method with several state-of-the-art SVM classification techniques that are capable of inferring multi-target screening models on three chemical data sets (cytochrome P450s, dehydrogenases, and a trypsin-like protease data set) containing three different biological targets each.

**Results:**

The experiments show that ranking-based algorithms show an increased performance for single- and multi-target virtual screening. Moreover, compounds that do not completely fulfill the desired activity profile are still ranked higher than decoys or compounds with an entirely undesired profile, compared to other multi-target SVM methods.

**Conclusions:**

SVM-based ranking methods constitute a valuable approach for virtual screening in multi-target drug design. The utilization of such methods is most helpful when dealing with compounds with various activity profiles and the finding of many ligands with an already perfectly matching activity profile is not to be expected.

**Electronic supplementary material:**

The online version of this article (doi:10.1186/s13321-014-0050-6) contains supplementary material, which is available to authorized users.

## Background

Considering the large potential market for drugs in some areas, like the treatment of CNS disorders [1], accompanied by a vast increase in R&D funding over the last years [2], one could argue that the struggle for new drugs should be crowned with success. However, the pharmaceutical industry yielded fewer therapeutic agents in relation to their expenses [2]. The confidence that drugs for single targets are capable of curing complex diseases, like cancer or diabetes, is deemed to be the cause of the aforementioned decrease in output [3,4]. The prevailing paradigm in rational drug design in the last decades could be described by the creation of selective ligands that are tailored to one target. Dealing with one target only helps to reduce side effects [5-7]. Albeit, the effectiveness of a drug is not only dependent on how well it can inhibit or activate its biological target. It is also a matter of how robust its underlying system is against perturbations [3].

In retrospective, selective high-affinity binders are an ideal case. In reality ligands effecting more than one target simultaneously are more likely to occur [8]. Especially when dealing with complex diseases, single-target drugs seem to be the wrong approach since the inhibition of individual proteins can be counteracted by additional signaling routes [5,7,9] and cross-talk [10,11], or the influence of therapeutic agents is compensated [3,12]. Based on the complex pharmacology of drugs against AIDS, cancer, metabolic and CNS disorders, selectively non-selective drugs tend to be more effective than a single high-affinity binder [1] because multiple associated processes have to be considered [13,14].

In recent decades multi-target drugs were more likely to be discovered by serendipitous events rather than knowing their promiscuity beforehand [7]. However, combining computational methods with the increasing amount of available bioactivity information should also yield compounds that express a certain activity profile with a higher likeliness [15]. According to Morphy et al. [6], it is unlikely to find a ligand during screening that already has all desired properties. Hence, there exist mainly three scenarios for the design of a multi-target drug: a) Two ligands that bind with a high affinity to distinct targets are used to “design in” a new ligand by uniting their structural elements responsible for activity. b) It is possible to identify a ligand that already shows a weak activity against the desired activity profile. In this case, the ligand has to be modified in order to increase its affinity. c) A multi-target ligand can also show an activity against an undesired target. Thus, a medicinal chemist has to “design out” the adverse binding properties [6]. Hence, one cannot rely on the identification of already perfect multi-target ligands during high-throughput screening. Instead it can be considered as beneficial to screen for molecules that partially fulfill the desired profile. These suboptimal ligands can then be built on to design a ligand with the appropriate activity profile.

The concept of multi-target drug design has multiple benefits compared to a drug cocktail made of various ligands or a multicomponent drug. When several molecules enter the human body the ADMET characteristics of each ligand have to be taken into account. Albeit, a multi-target drug accounts only for the absorption and elimination of a single molecule, which is a more manageable task [16]. Furthermore, a multi-target drug usually binds with a lower affinity to each of its targets in comparison to a single target drug [17], since the same key has to fit into multiple locks. Without the requisition of high-affinity binding, the process of multi-target drug design is not subject to the same high constraints as single-target drug design and therefore a higher amount of proteins can be targeted [13].

**Figure 1:**
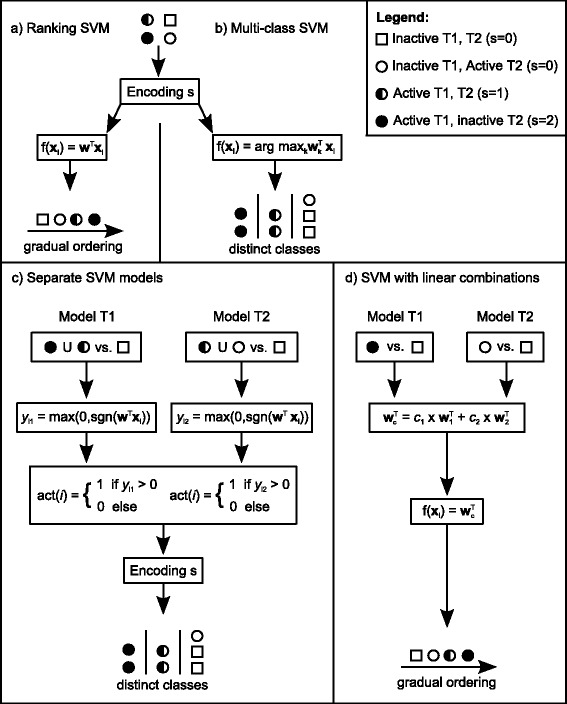
**Overview of the workflow of different methods for multi-target screening.** The example assumes a data set with a main target T1 and a secondary target T2 which should be avoided by the ligand. *S* *V* *M*_*Rank*_ **(a)** and the multi-class SVM **(b)** learn the encoding s of the activity profile. Separate SVM models **(c)** predict the activity of *i* via act(*i*) and infer distinct classes based on the proposed encoding. The SVM with linear combinations **(d)** uses subsets of the data set to build several models that are combined before prediction. The encoding s of the desired activity profile is reflected in the factors *c* of the linear combinations.

High-throughput screens (HTSs) are a valuable data source to infer predictive structure-activity relationships for virtual screening [18]. Standard machine learning methods such as Bayesian learning [19], neural networks [20], and support vector machines [21] (SVMs) are able to train virtual screening models on high-throughput data. The models can support the drug design process by facilitating the prediction of the sensitivity of a compound against a specific target. However, in multi-target drug design a lead candidate should have a desired sensitivity profile against a number of targets. If HTSs against additional targets based on the same combinatorial library are available, a machine learning method can take advantage of this data to include multiple sensitivity information in the virtual screening model. Ma et al. [22] combined separate sensitivity models for each of the targets to screen a database for compounds with a desired multiple-target profile (see Figure 1c). Their approach combined the prediction of separate sensitivity models to estimate the selectivity profile of a compound. However, separate models for individual targets are evaluated. Recently, large margin ranking SVMs [23] and structural SVMs [24] were introduced as valuable methods for virtual screening. Using a specific encoding, ranking methods can be applied to infer a model for a certain activity profile of compounds for different biological targets. In a study of Wassermann et al. [25] a ranking SVM was used for a SVM-based searching of target-selective compounds in order to discriminate between non-selectively active, selectively active, and inactive compounds for data sets with two targets.

The aim of this study is to present a machine learning framework based on large margin ranking methods, which is able to infer a model of target specific sensitivity information from HTSs against multiple targets. The method can be divided into two parts. First, we suggest a way to incorporate the data of HTSs against multiple targets into a single encoding. The resulting encoding represents the activity profile of a compound against a specific selection of targets and can efficiently be learned by the aforementioned ranking methods. Thus, in the second step, we employ a modified version of the linear ranking SVM SVM_Rank_ [26] to learn the encoding in a single machine learning model. We evaluated our method on a cytochrome P450 data set, and a dehydrogenase data set, both containing compounds with different activity profiles against three targets. Then we compared our results with a recent study of Heikamp et al. [27] about the linear combination of individual SVM models and the results of a multi-class SVM. At the end, we also evaluated the robustness of our method with respect to different activity cutoffs on a trypsin-like protease data set.

The results show that our method is able to infer machine learning models that incorporate sensitivity information from HTSs against multiple targets. In addition, compounds that do not completely match a respective activity profile are still ranked higher than entirely undesired profiles or mere decoys. To conclude, we think that the elaborated machine learning framework is a valuable tool to build models for screening compound libraries for molecules with a desired sensitivity profile and compounds with an almost matching activity profiles. Consequently, molecules that only partially fulfill the desired profile can then be modified by a medicinal chemist to comply with the given requirements.

## Methods

In this section we describe the linear combination of individual SVM models and the concept of the multi-class SVM (MC-SVM). Afterwards, we present the modified linear ranking SVM, which is able to learn various activity profiles. An overview of the methods is illustrated in Figure 1. Finally, we introduce the encoding that incorporates information from HTSs against multiple targets.

### Linear combinations

**Figure 2:**
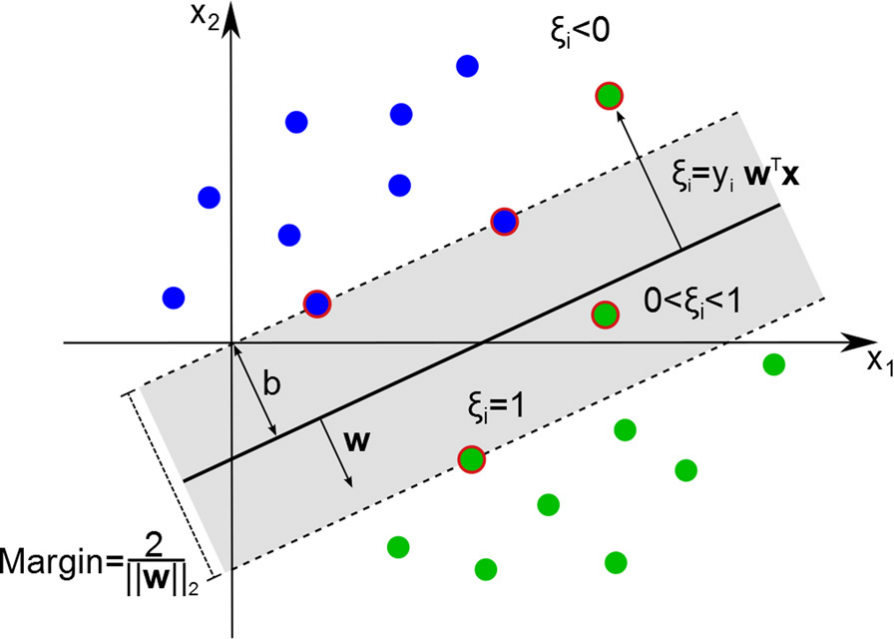
**Support vector classification (SVC).** Illustration of an SVC classification function represented by **w** ^*T*^ **x**. The slack variables *ξ*_*i*_=*y*_*i*_ **w** ^*T*^ **x** facilitate the trade-off between the size of the margin (indicated by a gray tube) and the error due to misclassifications. **w** denotes the weight vector, *y*_*i*_ the label of instance *i*, and **x** is the feature vector. *ξ*_*i*_ can assume a positive value between 0 and for 1 for training instances located in the margin. For instances on the wrong side of the margin *ξ*_*i*_ is less than 0. Support vectors are indicated by a red ring.

The concept of the linear combination of SVM models was introduced by Geppert et al. [28] and applied in a recent study of Heikamp et al. [27] to the prediction of active molecules with respect to overlapping activity profiles against multiple targets. This approach is based on a combination of the weight vectors **w** of separate SVM models to a single united weight vector **w**_*combined*_. Equation 1 shows the linear combination of *n* weight vectors, where **w**_*i*_ is the weight vector of the *i*-th model and *c*_*i*_ its linear factor. A single SVM model is described in Figure 2.

(1) $$ \mathbf{w}_{combined} = \sum\limits_{i=1}^{n} c_{i} \mathbf{w}_{i}   $$

The linear factor *c*_*i*_ of each model can attain a positive value to favor models representing desired properties or negative values to exclude undesired properties. Hence, the new model unites in its combined weight vector the facilitation of desired properties and strengthens the downgrading of undesired properties. To this extent the linear combination of SVM models is capable to rank compounds with overlapping activity profiles of *t*_*n*_ targets in a way that compounds with a certain profile receive a better rank than compounds that do not match a specific profile. An individual weight vector **w**_*i*_ is generated for each target *t*_*i*_ with known active compounds and decoys. Then, using a linear factor *c*_*i*_ for each weight vector, compounds can be ranked according to a desired activity profile (see Figure 1d). We employed the linear SVM of the LIBLINEAR library [29] for the implementation of this method.

### Multi-class SVM

Multi-class SVMs (MC-SVMs) are also able to learn the encoding of the different activity profiles by interpreting every possible rank score *s* as a separate class. As shown in Figure 1b, the class of an unknown compound **x**_*i*_ is then predicted by $f(\mathbf {x}_{i})=argmax_{k} \mathbf {w}_{k}^{T}\mathbf {x}_{i}$. Hence, MC-SVMs are able to include sensitivity information. However, MC-SVMs process no information about the rank order of the different classes. For example, swapping an inactive compound with an active, sensitive compound induces the same overall error as swapping a sensitive compound with a compound expressing an undesired activity profile. Additionally, in contrast to ranking algorithms, the output of a MC-SVM is the predicted class and not a gradual ordering.

We employed the linear MC-SVM of the LIBLINEAR library [29]. The implementation is based on the MC-SVM formulation of Crammer and Singer [30]. Unlike one versus all or all versus all strategies with binary classifiers, their formulation trains a multi-class SVM model based on a single optimization problem.

### Linear ranking SVM

Large margin ranking methods are valuable tools for solving information retrieval tasks such as virtual screening. With a suitable encoding, data sets with compounds of various activity profiles can labeled in a way that a ranking method can learn the different importance between them. Hence, we employed the linear ranking SVM SVM_Rank_ with a modified loss function to train the sensitivity encoding described in this paper.

**Figure 3:**
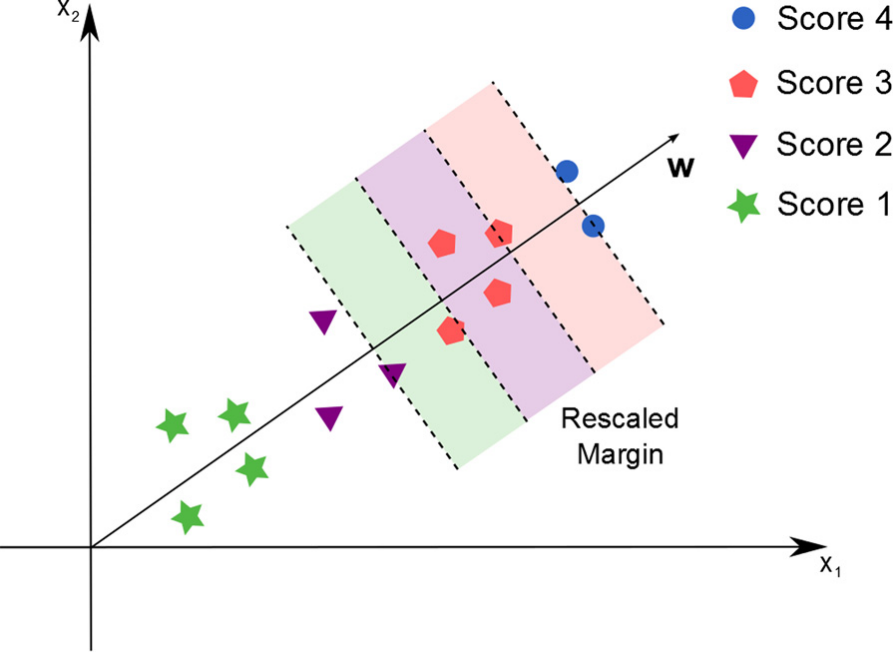
**Ranking SVM.** The learning algorithm of the ranking SVM yields a weight vector **w** that minimizes the pairwise loss dependent on the margin when the training instances are projected onto **w**. The overall ranking error is reduced to approximate the given ordering in the training set as effectively as possible along **w**. The principle of margin re-scaling allows for a ranking dependent on the degree of discrepancy in the ranking order and the pairwise loss is influenced by the *k*-partite ranking error. Therefore, ranking score 2 higher than score 4 is punished with a greater loss than a wrong order of the scores 4 and 3. This is indicated with an increasing margin dependent on the respective scores that are compared with each other.

SVM_Rank_ is based on the same structured SVM framework used by the virtual screening approach StructRank [24]. The main difference between both ranking methods is that SVM_Rank_ takes into account all ranks, whereas StructRank focuses on the topmost ranks. Thus, SVM_Rank_ optimizes the overall ranking performance (see Figure 3).

SVM_Rank_ learns a linear ranking function *f*(**x**)=**w**^*T*^**x**. The weight vector **w**=(*w*_1_,…,*w*_*n*_) is optimized such that *f*(**x**) is a large-margin function that minimizes the number of swapped ranks. The standard error function optimized by SVM_Rank_ is the fraction of miss-ranked pairs defined in Equation 2.

(2) $$ err_{rank}(D,f) = \frac{1}{|P|} \sum_{(i,j) \in P} loss(i,j)   $$

(3) $$ loss(i,j) = I_{\{f(\mathbf{x}_{i}) - f(\mathbf{x}_{j}) < 0\}} + \frac{1}{2} I_{\{f(\mathbf{x}_{i}) - f(\mathbf{x}_{j}) = 0\}}   $$

(4) $$ P = \{(i,j) | s_{i} > s_{j} \}   $$

*I*_*ϕ*_ is an indicator variable which takes the value one if the predicate *ϕ* is true and zero otherwise. *P* is the set of pairs that could be swapped. Loss(*i*,*j*) calculates the error if a pair (*i*,*j*) is miss-ranked by the ranking function *f*(**x**). $I_{\{f(\mathbf {x}_{i})-f(\mathbf {x}_{j})=0\}}\phantom {\dot {i}\!}1$ represents the case where two compounds with different ranks are assigned the same function value *f*(**x**). If the compounds are sorted by *f*(**x**), then an error occurs with a probability of 0.5. The ranking error function of Equation 2 has the disadvantage that it does not consider the difference between two scores *s*_*i*_ and *s*_*j*_. Thus, the original SVM_Rank_ cannot take into account and optimize the model for the different importance of the rank scores. To tackle this undesired behavior we used the *k*-partite ranking error (Equation 5) as error function for optimization.

(5) $$  err_{k-partite}(D,f)=\frac{1}{\sum_{(i,j)\in P} \left(s_{i} - s_{j}\right)} \sum_{(i,j)\in P} \left(s_{i} - s_{j}\right) loss(i,j)   $$

Clearly, Equation 5 includes the margin between the scores *s*_*i*_ and *s*_*j*_ and, thus, the different importance of the rank scores. The adjusted loss function can be readily integrated into the optimization problem solved by SVM_Rank_. The time complexity of the original SVM_Rank_ formulation is $\mathcal {O}(d \cdot l+R \cdot l+l \cdot log(l))$, where *l* is the number of training instances, *d* is the average number of non-zero features in the input vectors **x**_*i*_ and *R* is the total number of different scores *s*_*i*_ [26]. Including the *k*-partite ranking error in the linear ranking model requires an additional loop over the total number of different scores *R*. This additional loop results in a time complexity of $\mathcal {O}(d \cdot l+R^{2} \cdot l+l \cdot log(l))$. Hence, the algorithm is scalable to large chemical data sets depending on the average number of non-zero features *d* and number *R* of different rank scores *s*_*i*_. Both *R* and *d* should be kept as small as possible. Consequently, the number of targets is a limiting factor. Additionally, the molecular encoding used to encode the molecules should result in a sparse feature vector to reduce the dimensionality *d*.

The real-valued prediction of the linear ranking function *f*(**x**) allows a gradual ranking of all test compounds, which is a huge advantage compared to methods that only output class or rank values. A further advantage lies in the linearity of the ranking function *f*(**x**). The weights **w** of this function can potentially be interpreted and visualized in the same way as for linear SVMs. Therefore, it should be possible to represent the influence of each feature on the ranking order.

A feature with a high positive weight can be regarded as desired feature with respect to the provided rank scores, and compounds with such features are more likely to be ranked higher than other compounds. A negative weight implies undesired or unimportant properties at the lower end of the ranking. The recognition of the substructures related to a high rank is facilitated by representing the weight of each feature with a color code, as it was proposed by Rosenbaum et al. [31]. However, a feature with a high weight only corresponds to a high rank and not to a certain activity against a specific target.

### Encoding sensitivity for multiple targets

In this section we propose an example encoding for a hypothetical data set with three targets T1, T2, and T3 for a single- and dual-target activity profile. The chemical data sets used in this study are labeled on the basis of the described encoding.

A virtual screening against *m* multiple targets can be represented as a set *D* of *l* labeled fingerprints of compounds $(\mathbf {x}_{i},y_{i1},\cdots,y_{\textit {ik}},\cdots,y_{\textit {im}}), i = 1,\cdots,l,k = 1,\cdots,m,\mathbf {x}_{i} \in \mathbb {R}^{n}, y_{\textit {ik}} \in \{0,1\} $. Each compound *i* has a label *y*_*ik*_ for every target *k*. The label can be either inactive (*y*_*ik*_=0) or active (*y*_*ik*_=1). The goal to obtain sensitive lead candidates should be reflected by an encoding that incorporates information from HTSs against multiple targets. Hence, the target specific labels of a compound are encoded into a single label that can be utilized by a ranking method as the rank scores *s*_*i*_. The main idea of our encoding is to ensure sensitivity for a main activity profile while penalizing deviations from the desired activity profile. Consequently, compounds with almost matching activity profiles receive a high score whereas compounds with less desirable profiles obtain lower scores. Decoys are assigned the score zero.

In order to show that this approach is also valid for single-target drug design, we started our experiments with single-target activity. One target was regarded as main target with the highest priority and other activity profiles were labeled according to what extent they differ from the desired activity profile. To be more precise, the encoding of each compound reflects the number of labels *y*_*ik*_ that have to be changed from 0 to 1 or vice versa, in order to match the desired activity profile. Compounds that are not active for the main target were regarded as decoys regardless of their activity to the other targets. The precise labeling for the single-target activity is shown in Table 1. For the experiments assessing dual-target activity, the desired activity profile for two targets was regarded as main target with the highest priority. Other activity profiles were then labeled depending on how similar they are to the desired activity profiles. Compounds that also target the third undesired target were deprioritized (see Table 2). It is also possible to assign a different prioritization to each of the targets, which results in a slightly different ranking scheme. For single-target activity profiles, avoiding T2 could be more important than T3 (see Table 3). When screening for compounds with dual-target activity profiles for T1 and T2, getting compounds for T1 could be considered more important than for T2 (see Table 4).

**Table 1:** **Labels for a single-target T1**

**T1**	**T2**	**T3**	**Label**
1	0	0	3
1	1	0	2
1	0	1	2
1	1	1	1
0	1	0	0
0	0	1	0
0	1	1	0
0	0	0	0

As main target, T1 receives the highest value for its label. Other activity profiles are categorized on the basis of how strong they differ from the main activity profile.

**Table 2:** **Labels for a dual-target T1 and T2**

**T1**	**T2**	**T3**	**Label**
1	1	0	3
1	0	0	2
0	1	0	2
1	1	1	1
1	0	1	0
0	0	1	0
0	1	1	0
0	0	0	0

The activity profile for the two main targets T1 and T2 receives the label with the highest value. Other activity profiles are then categorized depending on to what extent they fulfill the desired activity profiles. Compounds that also target T3 are deprioritized.

**Table 3:** **Labels for a single-target T1 with deprioritization of T2**

**T1**	**T2**	**T3**	**Label**
1	0	0	3
1	1	0	1
1	0	1	2
1	1	1	0
0	1	0	0
0	0	1	0
0	1	1	0
0	0	0	0

As main target, T1 receives the highest value for its label. As for other activity profiles, avoiding T2 is more important than avoiding T3. Non-selective actives are regarded as decoys.

**Table 4:** **Labels for a dual-target T1 and T2 with prioritization of T1**

**T1**	**T2**	**T3**	**Label**
1	1	0	3
1	0	0	2
0	1	0	1
1	1	1	0
1	0	1	0
0	0	1	0
0	1	1	0
0	0	0	0

The activity profile for the two main targets T1 and T2 receives the label with the highest value. As for other single-target activity profiles, T1 is more important than T2. T3 has to be avoided. Non-selective actives are regarded as decoys.

In the study of Wassermann et al. [25] selective compounds were labeled with 1, decoys with 0, and non-selective active compounds with −1 in order to remove non-selective compounds for the higher ranks of a virtual screening. This was done for data sets with two targets each. In this study, decoys and compounds with completely undesired activity profile are assigned the label with the lowest priority. We think this more is appropriate for multi-target vHTS since even fingerprints of compounds that are non-selective still contain information why this compound is considered active for a specific target. Inactive compounds simply do not contain information for activity at all. This approach should result in a more precise ranking for multi-target vHTS and can also screen for compounds with slightly deviating activity profiles for further optimization. However, this applies only to well prepared data sets. If a data set contains highly promiscuous compounds against a broad range of targets, the features of such compounds rather interfere with the model than add additional information. The similarity-based methods described in this paper cannot distinguish between compounds for the desired targets and highly promiscuous binders once a data set is assembled. In case of doubt, such compounds should be discarded from the data set.

Our multi-target ranking method will be referenced as MT RANK and the ranking strategy of Wassermann et al. [25] as S RANK. For the weights of the linear combinations we always chose a linear factor of 2 for the desired activity profile and −1 for the models of the undesired targets to prioritize the main target(s).

From a machine learning perspective, the proposed encoding results in a ranking or ordinal regression problem. However, the different prioritizations of activity profiles induce a difference in the importance of the ordinals which is reflected by the margin between the possible scores *s*_*i*_. A machine learning algorithm trained on the proposed encoding should be able to learn this difference in importance.

### Molecular encoding

The molecular fingerprints used in this study were generated with the Java library jCompoundMapper developed by Hinselmann et al. [21]. We chose the common circular topological extended-connectivity fingerprint (ECFP) [32] to calculate the fingerprints for every compound in test and training sets. The use of ECFPs allows a fast comparison of molecules in an automated fashion. On the basis of the results of a previous paper from Rosenbaum et al. [33] we chose a bond diameter of 6 and a hash space of size 2^20^ bits as additional preferences for the hashed fingerprints. Further details can be found in the documentation of jCompoundMapper [21]. We generated functional-connectivity fingerprints (FCFP) with a bond diameter of 6 with CDK [34,35] in order to examine if they yield a similar performance. Detailed results of the FCFPs are not shown in this paper.

LIBLINEAR and the linear ranking SVM use the dot product kernel which generally increases the ranking error or reduces the AUC, since the fingerprints of larger molecules result in higher similarities. However, we normalized each fingerprint on the basis of its length, such that ∥*x*_*i*_∥=1. Consequently, an application of the dot product kernel to these normalized fingerprints is equal to the cosine kernel (see Equation 6). By this means the dot product kernel is normalized to [ 0,1] and thus is not influenced by the size of the fingerprints, which leads to better performance on chemical data sets in general.

(6) $$ k_{cos}(\mathbf{x}_{i},\mathbf{x}_{j})=\frac{\mathbf{x}_{i}^{T}\mathbf{x}_{j}}{\left\|\mathbf{x}_{i}\right\| \left\|\mathbf{x}_{j}\right\|}   $$

## Experimental

In this section we describe the chemical data sets and their preparation. Then, we present the experimental setup and parametrization of the algorithms used for the experiments.

### Chemical data

**Figure 4:**
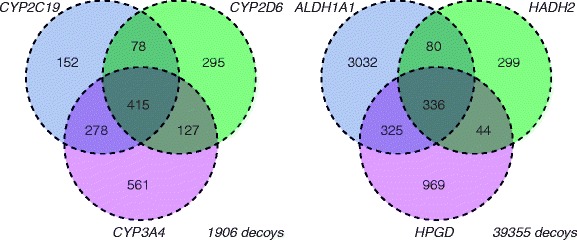
**Composition of the cytochrome P450 and dehydrogenase data sets.** Both Venn diagrams show the composition of the active compounds with respect to their activity profiles in the cytochrome P450 data set (left) and the dehydrogenase data set (right).

Based on the chemical data sets used in the study of Heikamp et al. [27] the same compounds were downloaded from PubChem’s BioAssay database [36]. As a result two data sets with compounds expressing single-, dual-, and triple-target activities from confirmatory bioassays were generated. The first data set comprises compounds with the aforementioned activity profiles for three biological targets of the cytochrome P450 family (CYP2C19, CYP2D6, and CYP3A4). The second data set consists of inhibitors for the three dehydrogenases aldehyde dehydrogenase 1 (ALDH1A1), hydroxyacyl-coenzyme A dehydrogenase type II (HADH2), and 15-hydroxy-prostaglandin dehydrogenase (HPGD). Figure 4 shows the composition of both data sets with respect to their activity profiles. The data set representing the cytochrome P450s contains 4807 compounds in total and the dehydrogenases data set 44440 compounds in total.

**Figure 5:**
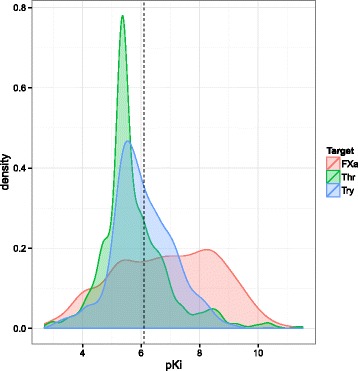
**Distribution of pK**_***i***_ **values of the trypsin-like protease data set.** This figure shows the distribution of pK_*i*_ values of the three trypsin-like protease targets Factor Xa (FXa), Thrombin (Thr), and Trypsin (Try). The initial activity cutoff is drawn as a vertical dashed black line at 6.1.

To investigate the behavior (e. g., robustness) of the described multi-target screening methods with respect to different activity cutoffs in the same data set, a trypsin-like protease data set was generated from BindingDB [37]. This data set comprises 881 compounds with pK_*i*_ values for Factor Xa (FXa), Thrombin (Thr), and Trypsin (Try). The distribution of the pK_*i*_ values is shown in Figure 5. Based on the description in Additional file 1, an initial activity cutoff of 6.1 was chosen. Since the pK_*i*_ values for FXa are more equally distributed than for Thr and Try, FXa was chosen as the single main target for this data set. In the same way the data set was processed for the activity cutoffs 5.6 and 6.6. A compound was regarded as selective for FXa, if both of the secondary targets have a pK_*i*_ value lower than the chosen cutoff.

To ensure an unbiased comparability of compounds in the chemical data sets the Standardizer, JChem 5.12.0, 2013, ChemAxon [38] (http://www.chemaxon.com), was used for each molecule in training and test set to canonicalize and transform every molecular structure. The standardization process was parameterized according to the guidelines of Fourches et al. [39]. As a result, each molecular structure was neutralized, tautomerized, aromatized, provided with explicit hydrogens, and underwent a two dimensional cleaning of its atom coordinates.

### Experimental setup

Similar to the study of Heikamp et al. [27] two experiments were carried out for the cytochrome P450 and the dehydrogenase data sets. At first, the algorithms were trained with a focus on compounds with a single-target activity. Then, the focus was changed to compounds that show dual-target activity profiles. The compounds of each data set were subdivided according to their activity profile. We sampled *n* compounds at random for training from each subset, whereby *n* denotes half of the smallest subset available for the same category (single, dual, and triple). Thus, each subset was limited by the size of the subsets from the same category. Each sampling was repeated 20 times. Compared to previous experiments with these data sets, we sampled 1000 decoys for each training set since linear SVMs allow for training an SVC model with a larger amount of instances. Regarding the trypsin-like protease data set, 20 training and test sets were sampled such that the distribution of labels was the same within both sets.

The linear ranking SVM can distinguish different activity profiles via a specific labeling of the training instances. Thus, compounds of each activity profile were included in each training set of SVM RANK and MC-SVM. Regarding the linear SVM with linear combinations (SVM LC), only compounds with the respective activity profile were used as active compounds in each training set as it was done in the original study. Each test set contained all remaining compounds that were not used for training.

The standard linear SVM, the linear ranking SVM, and the MC-SVM require the regularization parameter C for training. To determine a feasible value for C, a grid search with a 2-fold cross-validation was performed. Guided by a study of Agarwal et al. [23] we chose the range {0.1,1,10,100,1000} for the linear SVM as well as for the MC-SVM. The range {10^−6^,10^−5^,10^−4^,10^−3^,10^−2^} was selected for the linear ranking SVM. The specific labeling for the ranking SVM and MC-SVM was done according to the desired activity profile. All methods and evaluations were implemented in an in-house Java-based machine learning library.

For each training set the corresponding test set was labeled in two different ways to observe the performance on a test set with only the desired activity profile and on a test set with also deviating, yet workable activity profiles. At first, a binary labeling was used that reflected the activity to the main target(s) only. The second labeling met the same scheme that was used for the training sets of the linear ranking SVM. To this extent, the performance of the respective algorithms could be observed when screening for a single desired activity profile or when slightly deviating activity profiles are also relevant.

## Results and discussion

At first, we describe the validation of our experimental setup with data sets used in a study of Agarwal et al. [23]. Then, we present a comparison between the linear ranking SVM (RANK) and a standard linear SVM without linear combinations (SVM) on a simple binary labeling of training and test sets as baseline. Subsequently, we show and discuss the results of the three approaches MT RANK, SVM LC and MC-SVM on the chemical data sets with different activity profiles for single-target and dual-target activity with both a binary labeling and the proposed encodings of the test sets. Then, the results of the trypsin-like protease data set with different activity cutoffs are presented. The MC-SVM was only applied to the non-binary test sets, because it requires a multi-label classification problem. The binary test sets were also used to examine and compare the different ranking strategy of Wassermann et al. [25] (S RANK) for single-target activity profiles.

### Validation of the experimental setup

First, the performance of the linear ranking SVM was validated with data sets used in a study of Agarwal et al. [23]. In this validation the linear ranking SVM and the linear SVM without linear combinations were compared according to their AUC performance on a binary labeling. The given ranking error is equal to 1−*AUC*. We selected the five data sets CDK2, COX2, FXa, PDE5, and *α*_1*A*_AR provided by Jorissen et al. [40] with the four splits 1st/2nd, 2nd/1st, odd/even, and even/odd. We could observe that 15 out of 20 performance evaluations were in favor of the linear ranking SVM. This reflects the tendency that SVM-based ranking shows a better performance than SVM classification in general. Therefore, the implementation of the linear ranking SVM and the experimental setup can be regarded as sound.

### Comparison with standard linear SVM

**Figure 6:**
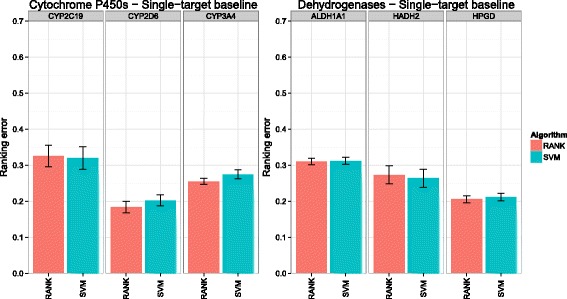
**Performance of the cytochrome P450 and dehydrogenase baseline data sets for single-target activity.** Each boxplot depicts the mean ranking error on the 20 randomly generated test sets for each target. The given ranking error is equal to 1−*A* *U* *C*.

**Figure 7:**
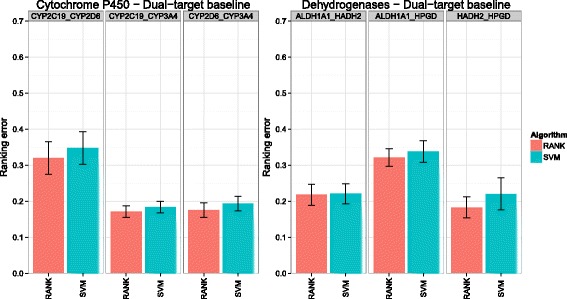
**Performance of the cytochrome P450 and dehydrogenase baseline data sets for dual-target activity.** Each boxplot depicts the mean ranking error on the 20 randomly generated test sets for each target. The given ranking error is equal to 1−*A* *U* *C*.

The results of the baseline data sets are depicted in Figures 6 and 7. As with all other data sets used in this study 20 training and test sets were sampled at random and the mean ranking error was calculated. The performances of the different baseline data sets are consistent with the results of Agarwal et al. [23] for a binary labeling of training and test sets. The linear ranking SVM can compete with the standard linear SVM and shows a slightly better performance for some targets, especially on the data sets for dual-target activity.

### Single-target activity

**Figure 8:**
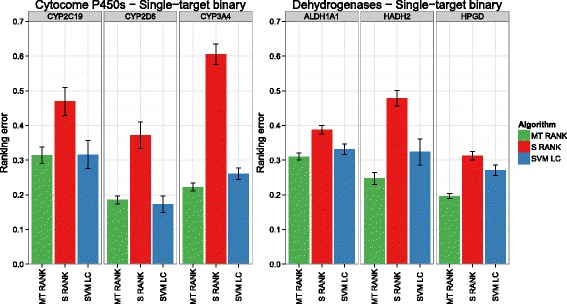
**Performance of the cytochrome P450 and dehydrogenase single-target data sets with binary test sets.** Each boxplot depicts the mean ranking error on the 20 randomly generated test sets for each target. The given ranking error is equal to 1−*A* *U* *C*.

Figure 8 shows the results of the training sets for single-target activity with the setup from Table 1 and a binary labeling of the test sets for the cytochrome P450s and dehydrogenases. The results show an overall better performance of the multi-target ranking method (MT RANK) compared to SVM with linear combinations (SVM LC). Furthermore, the higher ranking error of the selectivity ranking (S RANK) shows that this method has a negative influence on the recognition of single-target ligands. This method was originally applied to data sets with two targets only. A data sets with three or more targets contains also more non-selective activity profiles. Therefore, grading non-selective molecules lower than decoys can have an adverse effect. As said before, even non-selective ligands still contain activity information which can be utilized for a finely graduated ranking.

**Figure 9:**
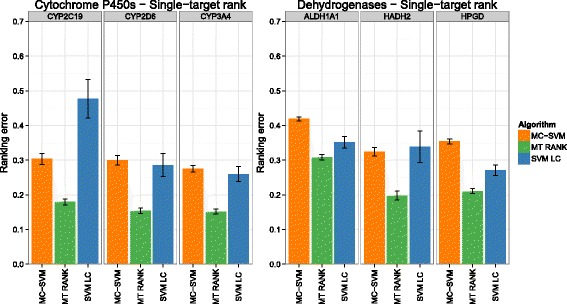
**Performance of the cytochrome P450 and dehydrogenase single-target data sets with ranking test sets.** Each boxplot depicts the mean ranking error on the 20 randomly generated test sets for each target.

Figure 9 presents the results of the experiments with the same training sets as in Figure 8, but with the elaborated labeling from Table 1 applied to the test sets. In this experiment the ranking error is not linked to the AUC because of the non-binary labeling of the test sets. The multi-target ranking method MT RANK outperforms SVM LC and the MC-SVM in each experiment. As to be expected, the graded labeling of the compounds results in a better ranking. The ranking method is able to learn the different importance between various activity profiles. Thus, ligands with a partially desired activity profile can be ranked higher than compounds with completely undesired profile.

### Single-target activity with different importance of secondary targets.

**Figure 10:**
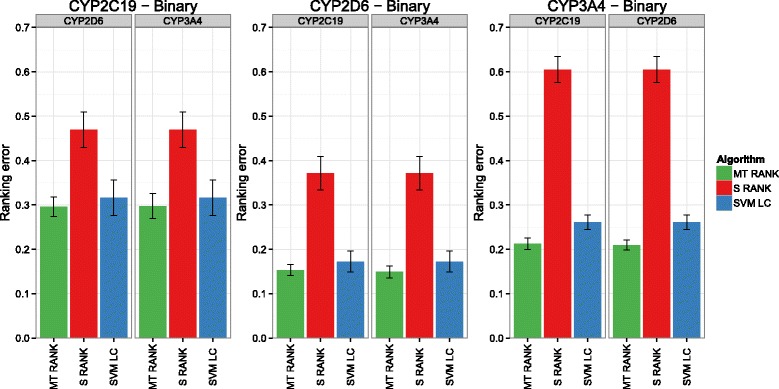
**Performance of the cytochrome P450s single-target data set with binary test sets.** Each boxplot depicts the mean ranking error on the 20 randomly generated test sets for each target separated according to the secondary target that should be avoided with higher priority. The given ranking error is equal to 1−*A* *U* *C*.

**Figure 11:**
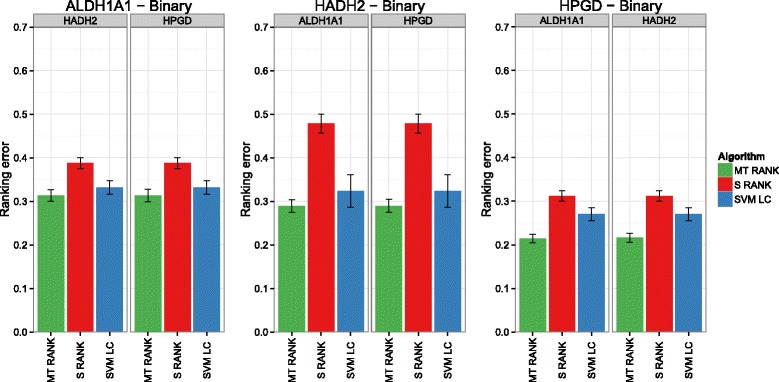
**Performance of the dehydrogenases single-target data set with binary test sets.** Each boxplot depicts the mean ranking error on the 20 randomly generated test sets for each target separated according to the secondary target that should be avoided with higher priority. The given ranking error is equal to 1−*A* *U* *C*.

**Figure 12:**
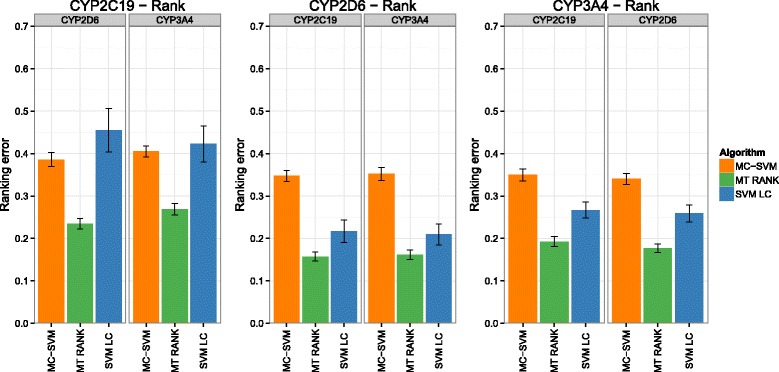
**Performance of the cytochrome P450s single-target data set with ranking test sets.** Each boxplot depicts the mean ranking error on the 20 randomly generated test sets for each target separated according to the secondary target that should be avoided with higher priority.

**Figure 13:**
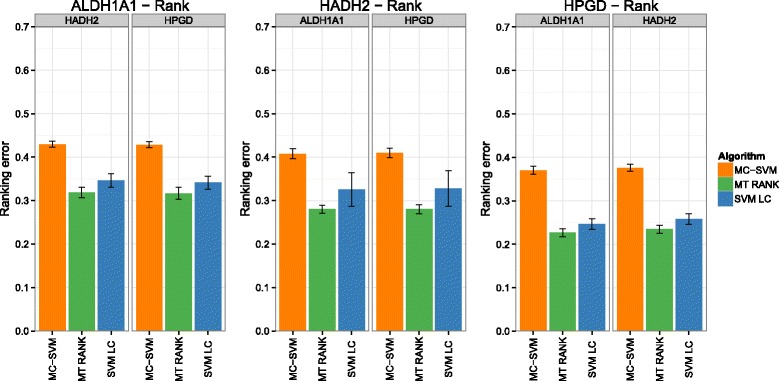
**Performance of the dehydrogenases single-target data set with ranking test sets.** Each boxplot depicts the mean ranking error on the 20 randomly generated test sets for each target separated according to the secondary target that should be avoided with higher priority.

In this section we show the results of the same experiments with the single-target activity profiles but with the alternative labeling from Table 3 applied to binary test sets (see Figures 10 and 11) and ranking test sets (see Figures 12 and 13). In this setup it was important to avoid one of the two secondary targets with higher priority than the other. The results show, that for the cytochrome P450 data set this ranking scheme is more beneficial regarding a binary classification than the one described in Table 1. Treating non-selective actives as decoys with a label of 0 seems to improve the ranking performance. The results of the ranking test sets show, that avoiding one secondary target with higher priority is more demanding for MT RANK and MC-SVM. However, the ranking SVM still shows a better performance in general. Changing the linear factors for the linear combinations to +3 for the main target, −2 for the secondary target to be avoided with higher priority, and −1 for the remaining secondary target did not show any significant improvement in performance for SVM LC.

### Dual-target activity

**Figure 14:**
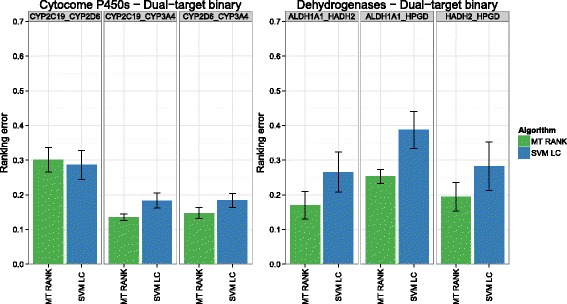
**Performance of the cytochrome P450 and dehydrogenase dual-target data sets with binary test sets.** Each boxplot depicts the mean ranking error on the 20 randomly generated test sets for each target. The given ranking error is equal to 1−*A* *U* *C*.

**Figure 15:**
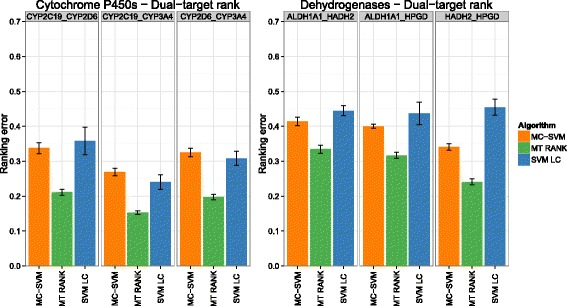
**Performance of the cytochrome P450 and dehydrogenase dual-target data sets with ranking test sets.** Each boxplot depicts the mean ranking error on the 20 randomly generated test sets for each target.

The results of the experiments with the dual-target activity profiles are depicted in Figure 14 for the binary test sets and in Figure 15 for the elaborated labeling described in Table 2. Once again, the results are in line with the aforementioned findings. The multi-target ranking method generally shows a better performance except for CYP2C19_CYP2D6 with a binary labeling of the test sets. Regarding the experiments with the dehydrogenases data set, the SVM with linear combinations features a lower performance compared to MT RANK. This behavior may derive from the fact that the SVM with linear combinations has more problems when there are few compounds for the desired label, since the multi-target ranking can compensate this issue with the training instances of similar activity profiles. Approaches that are based on individual models train independent models for each respective activity profile. MT RANK uses all activity profiles to learn a model in one step and therefore is less prone to fewer training instances, since activity profiles deviating from the main target still contain information about their targets. Another reason can be the imbalance between dual-target training instances and training instance for the respective undesired 3rd target, which does not seem to be a problem for the multi-target ranking method. The results for the non-binary labeling also show a lower ranking error for MT RANK compared to SVM LC and MC-SVM as could be observed in the results of the single-target data sets.

### Dual-target activity with different importance of the main targets.

**Figure 16:**
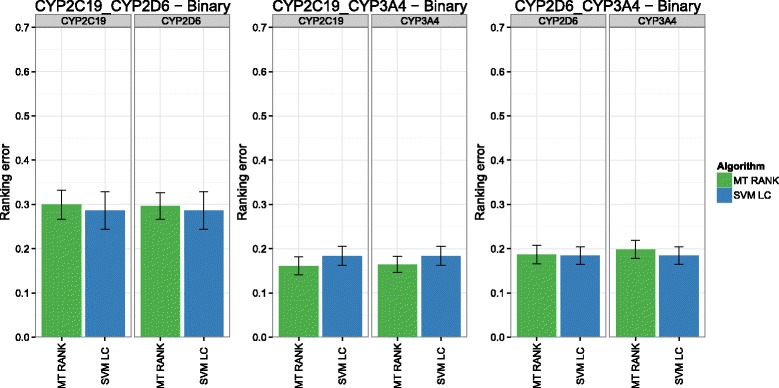
**Performance of the cytochrome P450s dual-target data set with binary test sets.** Each boxplot depicts the mean ranking error on the 20 randomly generated test sets for each target separated according to which of both main target should be regarded with higher priority. The given ranking error is equal to 1−*A* *U* *C*.

**Figure 17:**
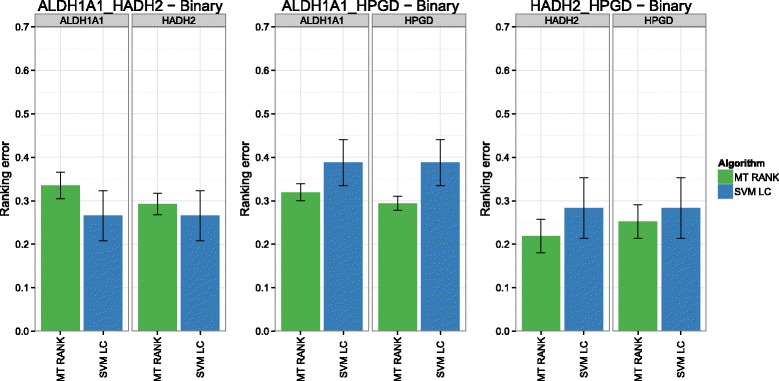
**Performance of the dehydrogenases dual-target data set with binary test sets.** Each boxplot depicts the mean ranking error on the 20 randomly generated test sets for each target separated according to which of both main target should be regarded with higher priority. The given ranking error is equal to 1−*A* *U* *C*.

**Figure 18:**
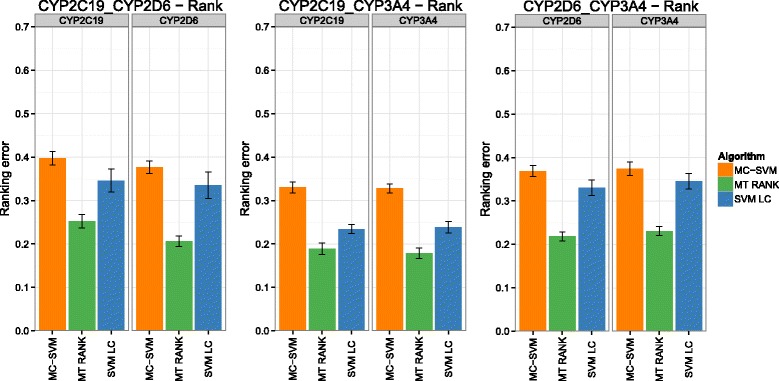
**Performance of the cytochrome P450s dual-target data set with ranking test sets.** Each boxplot depicts the mean ranking error on the 20 randomly generated test sets for each target separated according to which of both main target should be regarded with higher priority.

**Figure 19:**
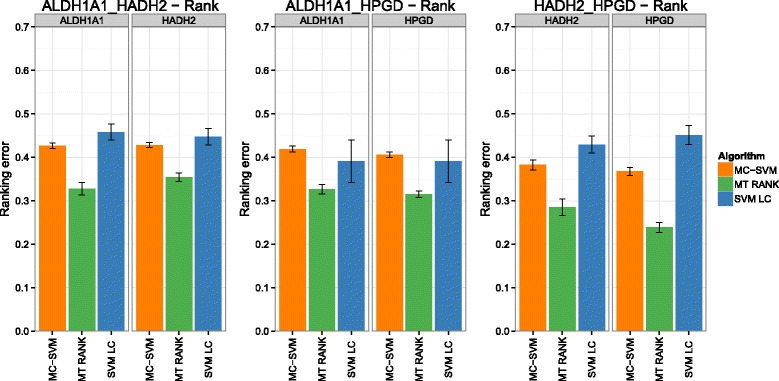
**Performance of the dehydrogenases dual-target data set with ranking test sets.** Each boxplot depicts the mean ranking error on the 20 randomly generated test sets for each target separated according to which of both main target should be regarded with higher priority.

The Figures 16, 17, 18 and 19 show the results of the same experiments with the dual-target activity profiles but with the alternative labeling from Table 4. In this experiment single-target activity profiles for the first of the two main targets were regarded as more important than activity profiles for the second one. The results of both the binary and the ranking test set show, that the ranking scheme of Table 4 is less optimal for MT RANK and MC-SVM than the one described in Table 2. When screening for a dual-target activity, a different prioritization of both main targets seems to be more challenging for the ranking SVM.

### Trypsin-like protease data set

**Figure 20:**
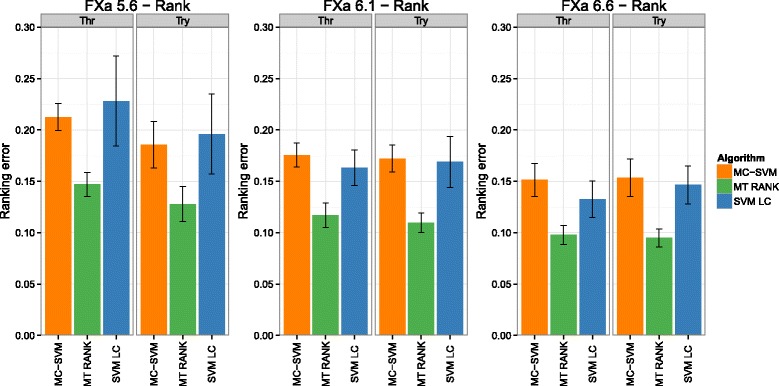
**Performance of the trypsin-like protease data set with FXa as main target.** Each boxplot depicts the mean ranking error on the 20 randomly generated test sets for each target separated according to the selected activity cutoff and which of the secondary targets should avoided with higher priority.

The chosen cutoffs 5.6, 6.1, and 6.6 combined with the labeling of Table 3 resulted in the different distributions of labels that are shown in Table 5. An increasing activity cutoff results in more selective compounds for FXa and fewer compounds that also target one of the secondary targets. Figure 20 shows the performance on the trypsin-like protease data set with the three different selectivity cutoffs. Despite of the selected activity cutoff, the linear ranking SVM has a lower ranking error than SVM LC and MC-SVM. However, with a decrease in compounds that are also selective for one of the secondary targets the advantage of MT RANK slowly diminishes. This behavior simply derives from the fact, that there are less and less ranking errors to be made.

**Table 5:** **Distribution of labels in the trypsin-like protease data set for different activity cutoffs**

	**Percentage of compounds with label**			
**Cutoff**	**3**	**2**	**1**	**0**
5.6	15	25	5	55
6.1	25	23	4	48
6.6	32	17	3	48

In this overview avoiding Thr is more important than Try. In the reverse case the values for the labels 2 and 1 just have to be switched. The activity cutoff is given in pK_*i*_.

### Influence of the importance of different activity profiles

The results of the experiments with single and dual-target activity indicate that the SVM with linear combinations has plainly more problems ranking similar activity profiles higher than mere decoys. However, this fact is not unexpected, since the SVM LC does not optimize the ranking error during training. In addition, the MC-SVM with its distinct classes shows also a higher ranking error compared to the multi-target ranking approach. This issue is supported by examining the ranking error (results not shown) among the active compounds only and ignoring all decoys completely. SVM LC and MC-SVM have more difficulties to rank compounds similar to the main activity profile higher than decoys. Furthermore, experiments show that increasing the difference in importance between the active compounds accounts for a decrease in the ranking error. In this setup, the *k*-partite ranking error enforces a more strict ordering of active compounds with similar activity profiles.

A comparison between additional ranking experiments (results not shown) led us to the conclusion that treating completely non-selective compounds as decoys with a label of 0 can in general be considered beneficial for the ranking SVM. Especially, when their activity can be related to non-specific events like protein binding or aggregation, non-selective compounds should not be more important than decoys. However, the lower performance of the binary classification of HADH2 in Figure 11 indicates that in some cases valuable information can even be contained in non-selective compounds if their activity is not related to non-specific events. Removing non-selective compounds entirely from the training set instead of keeping them as decoys did not influence the performance significantly.

### Difference between ECFPs and FCFPs

The results of the experiments with FCFPs show that the performance of MT Rank and MC-SVM on the cytochrome P450s data set is quite similar. In general, the ECFP works slightly better with both methods. However, SVM LC shows a slightly better performance for the targets CYP2C19_CYP3A4 and CYP2D6_CYP3A4. The ranking error of SVM LC was significantly higher for CYP2C19 and significantly lower for CYP2D6. Regarding the dehydrogenases data set, it is noticeable that the FCFP shows a significantly higher ranking error for each method. Nevertheless, the overall ratio in performance between the methods did not depend on the choice of the fingerprint.

## Conclusions

To conclude, we think that the proposed encoding in combination with *SVM*_*Rank*_ is able to handle compounds of different activity profiles in a way that compounds not fitting the desired activity profile to 100% are still part of the higher scoring compounds in vHTS. Thus, each activity profile can still be included in a virtual screening model without losing information that is characteristic for each specific activity profile. Especially, when already perfectly matching ligands for a certain activity profile are not to be expected in sufficient numbers in a vHTS, a medicinal chemist can amend suboptimal compounds to suit certain requirements. Therefore, a ranking SVM can be considered a valuable approach in multi-target vHTS because it directly solves the actual problem in form of a small ranking error for a specified problem instead of the ranking derived from a classification model. The specific problem encoded in the labeling of each data set is not fixed but highly variable. The user can choose the labeling that fits him best for his problem. To this extent, different criteria can influence the labeling and the same activity profiles can be treated differently depending on the task at hand. But even if a more diverse composition of the upper ranks of a virtual screening is not desired, our approach is still feasible for a simple virtual screening as can be seen from the experiments with the binary test sets.

In comparison to other methods, the ranking performance is also robust against the chosen activity cutoff, as demonstrated with the trypsin-like protease data set, as long as there are enough compounds with different ranking scores in the data set. Nevertheless, similarity-based virtual screening methods are only as good as the data set they are applied to. They can assist in the drug design process and can speed up lead identification, but their prospective results still have to be validated experimentally since they strongly rely on the applicability domain (AD) of the respective data set. Therefore, it is quite difficult to examine a different AD and a divergent region of the chemical space other than the one the data set was provided for. If the compounds of a data set are structurally too similar, the finding of entirely different scaffolds for the same binding pocket is not to be expected.

A focus in future studies might be to redesign this method to focus on a single main target and include selectivity information of secondary targets in form of different pK_*i*_ values. Therefore, lead candidates for a specific target can be identified that also have a desired selectivity profile against a number of secondary targets. It is possible that the activity against a desired target is caused not by the target itself but rather by an activity at another downsteam or upstream location. This can be a problem for all multi-target methods and has to be assessed in further studies. The exclusion of compounds for some assays regarding this problem could be beneficial for the performance of multi-target methods. Furthermore, the methodology of transductive SVMs [41] could be used to enable training on data sets with missing labels. The principles shown in this paper could be used in future studies to calculate the importance of a feature for the activity against a specific target.

## Additional file

Additional file 1
**The trypsin-like protease data set.** This document gives additional details on the composition of the trypsin-like protease data set from BindingDB.
