# Supplementary material for: A ranking method for the concurrent learning of compounds with various activity profiles
Source: J Cheminform. 2015 Jan 16;7:2. doi: 10.1186/s13321-014-0050-6 (PMC4306736; doi:10.1186/s13321-014-0050-6)
Supplement: Additional file 1 — The trypsin-like protease data set. This document gives additional details on the composition of the trypsin-like protease data set from BindingDB. [file 13321_2014_50_MOESM1_ESM.pdf]

# The trypsin-like protease data set

This document gives additional details on the composition of the trypsin-like protease data set from BindingDB [1].

## Preparation

This data set was compiled by the authors from compounds of the binding affinity database BindingDB. The data set comprises $pK_i$ values for each of the trypsin-like proteases factor Xa (FXa), Thrombin (Thr), and Trypsin (Try).

The data set was prepared as follows. First, we downloaded all molecules that contained $pK_i$ information for each of the three proteases yielding 886 molecules in total. However, many of these compounds were labeled with ambiguous $pK_i$ values. Consequently, we reconciled the database $pK_i$ values with the values in the original publications and resolved ambiguities (e. g., separate $pK_i$ values for +/- enantiomers of a compound). In this preprocessing step, one compound was removed from the benchmark data set because no sensible $pK_i$ value could be chosen. After this filtering step 881 compounds remained.

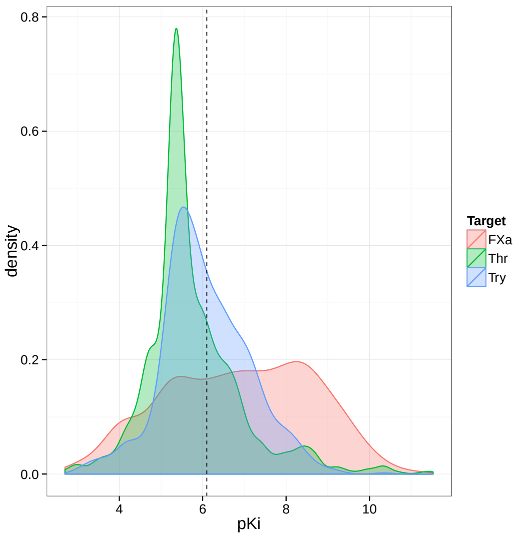


Figure 1: **Distribution of $pK_i$ values of the trypsin-like protease data set.** This figure shows the distribution of $pK_i$ values of the three trypsin-like protease targets Factor Xa (FXa), Thrombin (Thr), and Trypsin (Try). The initial activity cutoff is drawn as a vertical black dashed line at 6.1.

## Activity cutoff

To make this data set suitable for our encoding we had to define a cutoff for labeling a compound inactive ($y_{ik}$=0). We chose to set the cutoff to the mean of the medians of the $pK_i$ values of the three protease targets. This calculation resulted in a cutoff of 6.1. We visualized the cutoff and the distribution of the $pK_i$ values in Figure 1. While the $pK_i$ values against FXa are equally distributed in the range of 5.5 to 8.5, the $pK_i$ values against Thr and Try are

crowded around a $pK_i$ of 5.5. This difference in $pK_i$ value distributions can be observed because most of the original publications optimized the potency against FXa. The calculated cutoff is slightly larger than the $pK_i$ value density peaks of the secondary targets Thr and Try. Thus, we think that a cutoff of 6.1 is a feasible value.

# References

[1] Tiqing Liu, Yuhmei Lin, Xin Wen, Robert N Jorissen, and Michael K Gilson. Bindingdb: a web-accessible database of experimentally determined protein–ligand binding affinities. *Nucleic Acids Res.*, 35(suppl 1):D198–D201, 2007.
